# Variola in Buffalo

**Published:** 1882-03

**Authors:** J. I. Marcley


### BUFFALO

### Medical and Surgical Journal.

Vol. XXI. MARCH, 1882. No. 8.

### Original Communications.

### Variola in Buffalo.

By J. I. Marcley, M. D.

The following cases of small-pox occurred in my practice at Black Rock, during the period of my service as assistant to the Health Physician. As it is a disease seldom met with, a short history of the cases may be of interest to the profession.

The first case was that of Lewis Schreiber, who was taken sick on the 15th of November, 1881. Whether this was the beginning of the fever or of the eruption, I am unable to say. He was attended by a "Rational" Doctor, one Von Schulenburg. The case was diagnosed as one of scarlet fever. It was *not* reported as small-pox.

This young man's father was taken sick on the 4th of December, and was also attended by the same party. The father died, with symptoms of acute œdema of the glottis, on the 10th, after a sickness of six days' duration.

On the 7th of December, Albert, aged six years, was taken sick; I first saw the family on the 11th; I found the father dead in the house; Lewis, the first affected, was then well; decrustation being almost completed; but from the great number of pigmented maculæ on the *face*, breast and arms, and later from the pock-marks resulting, the case must have been of the coherent form on those parts mentioned.

Albert was then in the beginning of the fifth day of the disease, the third of the eruption; disease was of the coherent form, which terminated in recovery. All three had been successfully vaccinated in infancy. In this house were three other persons who escaped, viz., mother and two children aged 18 and 9 years, all vaccinated in infancy only. From this family arose all the other cases, and all but one can be traced directly to them.

Where the Schreibers contracted the disease I was unable to learn; they claim that none of them were away from home, and no visitors had been to see them. The boy Lewis was employed in the Malleable Iron Works up to the time of his sickness.

The next case was that of Mary Koch, aged 9 years, a cousin of the Schreibers, at whose house she had been visiting during the time of Lewis' sickness. She was taken sick on the 14th of December, with the ordinary symptoms of fever, headache, vomiting, etc.; the disease proved confluent in form and terminating in recovery; she had been vaccinated only once, about four months previous, *not* successfully.

Lewis Koch, aged 6 years, a brother of the above, began at the same time with similar symptoms; never vaccinated; disease confluent, ending in recovery.

Katie Koch, aged 3 years, a sister of the above, was taken sick on the 21st of December, disease being confluent; never vaccinated; recovery.

Adam Koch, aged 39 years, father of these children, began on the 21st of December; the disease mild varioloid; vaccinated once when a child. In this house, and in constant contact with the sick ones, were Mrs. Koch, aged 38 years, vaccinated only once, that in infancy, and two other children aged 12 years, and an infant of six months. The former was vaccinated when an infant, and the latter not until the disease was discovered in the family, and then it proved very successful; these escaped.

Wm. Metz, aged 7½ years, was the next one discovered, who, although he had never been in the Schreiber house, was a constant playmate of young Schreiber during the time his elder brother was sick. Willie began on the 22d of December with sore throat, chills, fever, headache and vomiting. The eruption appeared on the 25th, proved confluent in form; never vaccinated; recovery slow; decrustation not completed until the end of the fifth week.

Albert Metz, father of the boy Willie, aged 31 years; began on January 8th with chills, high fever, headache and backache, eruption not appearing until beginning of the fifth day, then only a few spots, constituting a mild case of varioloid. Mr. Metz was successfully vaccinated when an infant; I re-vaccinated him as soon as the disease was discovered in his son, but it did not "take;" Mrs. Metz, his wife, who escaped, I re-vaccinated at the same time, it taking very nicely. The virus used in each case was perfectly fresh, ivory points, prepared by myself from a cone obtained from the New England Vaccine Co., whose virus I have found to be the most reliable of any stored virus I have used. I think it proves as often successful as in the arm to arm method.

Jacob Stinner, aged 19 years, came next. After the death of Mr. Schreiber, and before the nature of the disease became known, Mrs. Schreiber took some dress goods to the Stinner boys' mother, who is a dressmaker, to be made up. The goods were taken to her on Saturday the 10th; on Sunday, the 11th, the disease was pronounced small-pox in the Schreiber family. Mrs. Stinner at once returned the goods, but not until after her son had contracted the disease. He was taken sick on the 22d of December with fever-chills and very severe headache, the eruption appearing on the afternoon of the 25th, and proving coherent in form, terminating in recovery. He had been successfully vaccinated once in infancy.

Lewis Stinner, aged 14 years, a brother of the above, developed symptoms of the disease about twelve days later, it proving to be very mild varioloid, not over a dozen spots appearing in all. This boy had been vaccinated more than once successfully. In this house, and in more or less constant contact with the patient, were Mrs. Stinner, who had varioloid when a child; Miss Stinner, aged about 24 years, vaccinated eight years ago and who had a "very sore arm" at that time; also two other children, aged about ten and twelve years. The eldest had been successfully vaccinated once; the youngest not until a day or two after her brother was taken sick, when I vaccinated the whole family. None "took" however, except this one. All escaped.

The next case was that of Dr. C. G. Champlin, who was taken sick on Sunday, the 25th of December. The Doctor was so sick during the initial fever, with symptoms resembling severe typhoid, that his life was despaired of, but when on Tuesday, the 27th, the eruption appeared, his condition at once improved, and the disease proved to be not very severe varioloid, running its course in a short time and without special trouble. Dr. C. was exposed to the contagion at the Schreiber house on Sunday, the 11th of December. He had been successfully vaccinated when a child, and after exposure it was repeated three different times, but did not take, although the virus was good, as proved by the fact that his brother was successfully vaccinated at the same time. His sister, who was in constant attendance on him, was successfully vaccinated and escaped the disease.

Frederick Roesch, aged 45 years, sat up with Mr. Schreiber the night he died. He was taken sick on the evening of the 23d of December; complained of headache, nausea, chills, fever, and intense backache; this was the only case in which backache was very severe. The eruption appeared on the 26th; with the appearance of the eruption, active delirium came on, and on the night of the 27th the patient escaped from his nurse and ran out on the street, through a sharp rain, and in no other clothing than a shirt. For some days after this it was necessary to have two men in attendance to restrain him; disease confluent in form; the eruption ran its course normally up to the seventh day, when the pocks began to change color, growing of a reddish hue; in places where there were no pocks, petechiæ appeared, gradually changing to a deep purple over the whole of the body, face and extremities, except the hands and feet, which were of a coal-black color. The pulse became slow (40 per minute) and very weak and irregular; tongue dry and cracked; sordes formed upon the teeth; the gums were swollen and bled easily; the mouth remaining partly open; delirium now became less active, and assumed a low muttering character; patient in a semi-comatose state all the time; liquid food and medicine were swallowed with difficulty when introduced into the mouth.

The treatment of this case was mainly tonic and stimulant, consisting of quinine, ergot, morphia, beef tea and milk. Whiskey in form of strong egg-nog being administered every halt hour, night and day, for some time; patient made a slow and tedious recovery; decrustation not complete until sixth week. Roesch was successfully vaccinated once, about twenty-five years ago.

In this house, and in more or less constant contact with the sick man, were Mrs. Roesch and five children. The wife was once vaccinated in infancy; the children all successfully within four years, with the exception of one which had not been done in six years. I re-vaccinated them all, only one taking, the last mentioned; *all remained free from the disease.* This case illustrates the protective power of recent vaccination; these people it was impossible to isolate, all the children being with their father a good portion of the time.

Jacob Brown, aged 47 years, in company with Mr. Roesch, sat up with Schreiber the night he died. On the 23d of December he complained of chills, fever, headache, pain in abdomen, nausea and slight backache. The eruption appearing on the 27th (four days from the commencement of the fever) and proving to be confluent; rather active delerium began as soon as the eruption was in full bloom, lasting for several days; decrustation took place slowly, face not clean at end of fifth week. In this connection I will state that in only one or two cases did the face clear off first. The rule was body and limbs first, hands and face several days later. In all these cases the eruption appeared simultaneously upon the face and arms. In all cases it could be early noticed in the mouth, especially on the roof just anterior to the soft palate. In two of my cases the eruption was very severe in the mouth, throat and nasal passages. And in all cases it appeared there to a slight degree, the inside of the cheeks and tongue being free from eruption in most all cases.

Mr. Brown had been vaccinated once when a child. In this house were Mrs. Brown and one son, aged 20 years. Mrs. B. was successfully vaccinated at the age of 18; the son when a small child, again at the age of 14, and at this time by myself; each time successfully; both escaped.

Mrs. John G——, aged 26 years. In this case I have been unable to trace the point from which they received the contagion, but there can be no doubt that it arose from the one common source. There is no evidence of Mrs. G's ever having been vaccinated, nor can she remember of its having been performed. Mrs. G. was pregnant between three and a half and four months. On the 23d of December she complained of being chilly; had headache and some backache, and nausea. On the morning of the 27th the eruption appeared, at which time she was taken with labor pain and hæmorrhage, followed soon by the expulsion of the contents of the uterus. The disease proved to be of the confluent form, and coherent over most all the body. At no time during the normal period did the lochia cease. Mrs. G. made a good recovery; desquamation not completed until fifth week. In this house Mr. O., aged 65 years, who had been vaccinated when a child, and who was in constant attendance on the sick, escaped the disease.

John G., aged 29 years, husband of Mrs. G., was taken sick on the 10th of January, fourteen days from the time eruption appeared on his wife. Symptoms were fever, chills, headache, backache and very severe and almost incessant vomiting; for this I prescribed 1-32 grain iodide of potassium with one drop of wine of ipecac, hourly. This gave great and almost immediate relief, stopping the vomiting after two or three doses.

Mr. Ghruler was vaccinated when a child, and again as soon as his wife's disease was diagnosed, but at that time it did not take. Disease of confluent type; throat, mouth and nasal passages were filled with the eruption; patient made a fair recovery.

John Staubits, aged 48 years, vaccinated successfully when a child; disease of the distinct form and mild; recovery good. Mr. S. was in the habit of stopping at a certain saloon on his way home from work where the Schreibers frequented; here it is, in my opinion, that he contracted the disease. In this house were his wife and one son, aged 13 years; both had been successfully vaccinated in childhood and both escaped the disease.

Mrs. Chas. Steinhauser, aged 36 years, a daughter of the man Schreiber, was at her father's house for about three hours the morning he died; was taken sick December 22d, twelve days after exposure, with a mild form of varioloid, so mild in fact that they kept the case to themselves, her husband continuing at his work until by some means the report was noised about that his wife had small-pox. I visited the house on the eighth day of her disease and put up the usual sign. In this house were her husband and two young children. The man had been successfully vaccinated when a child and escaped. One child, a boy aged 4 years, was taken sick on the 2d of January; complained of headache and vomited towards night, but still continued to play around; on the morning of the 3d, at about 9 o'clock, he had a severe convulsion lasting for over an hour; at 11 A. M. it was repeated, ending in death in about half an hour; no eruption appeared; this child had never been vaccinated.

The baby aged 6 months was also taken sick on the 2d, and on the 4th the eruption began to appear. Although this child had never been vaccinated, the disease was mild of the distinct form, coherent only around the neck in places, and the child made a good recovery.

George Hohl, aged 11 years, was the last case. He began January 1, '82, with headache, vomiting, etc.; eruption appeared on the 4th; disease confluent, considerable throat trouble; good recovery. This boy had been vaccinated a number of times, but never successfully. In this house were the parents of the boy, both of whom had been vaccinated in childhood and both escaping. There were also three children, a girl aged 19, never successfully vaccinated, although it had been tried a number of times and twice by myself with perfectly fresh virus; one girl, aged 18, vaccinated successfully five years ago, and a boy, aged 12 years, never successfully vaccinated. They were all in contact with the sick boy and all escaped.

This completes the list. I have tried to give the main points of interest in each in as short a manner as possible. I will now give a brief summary of some of the interesting and instructive features of these cases.

First, in regard to the occurrence of the different symptoms.

Chills were present in all cases, in some quite severe.

Headache of a more or less violent character was present in all.

Backache occurred in ten, but was violent in only one, the hæmorrhagic.

Nausea was present in all; vomiting in eleven; violent and long continued in only one case.

The tongue was much coated in all cases, but was not the seat of much eruption, only in two.

Sore throat was complained of in eight, severe in only two.

Delirium was present in four, very violent only in the hæmorrhagic case.

Abdominal pain in one case.

Constipation was marked in all cases; convulsions occurred in only one case.

Itching was distressing in two or three only.

Conjunctivitis was present in four cases, severe in only one.

Pain in hands and feet was complained of in six cases, severe only in the hæmorrhagic.

Œdema of lower extremities was marked in only one case.

Loss of hair occurred only in one, to any extent.

Pulse, owing to the difficulty in getting at this symptom, on account of swelling, it was neglected.

In regard to the period at which the eruption appeared in those cases that I could follow closely, it varied from two to five days from the beginning of the initial fever.

The period of incubation was twelve days in seven cases, and in three fourteen days. These were all I could actually date.

In regard to vaccination; thirteen had been vaccinated, none, however, within a number of years. Eight had never been vaccinated. Of those vaccinated the disease assumed the form of varioloid in five cases. Distinct in one, coherent in four, confluent in two, and hæmorrhagic in one case.

Of those not vaccinated, distinct in one, confluent in six. One died before the eruption appeared. In only one case was there any history of a successful (or non-successful) re-vaccination. This one had the disease the lightest of any, only a dozen spots appearing.

Although these cases are few in number, they speak very strongly in favor of vaccination and especially re-vaccination.

I have experienced a great deal of opposition among the German people to vaccination. This seems strange, for it is very seldom you will ever find a person, born in Germany, who has not been vaccinated, as there it is compulsory. The moment they land on our free soil, they imbibe the spirit of freedom, especially as regards vaccination.

The treatment of these patients was very simple. Anodynes (in form of morphia or Gregory's bromated syrup of Dovers, which I found very good) and cathartics, or rather laxatives and stimulants, as required. Potass chloral was found very good in the sore throat of this disease. Locally I found nothing so agreeable to the patient as olive oil, and as far as preventing the pitting goes, I do not see but it proves as satisfactory as anything else.

The method followed in disinfection, was the burning of sulphur in tightly closed rooms, leaving them so for several hours, then thoroughly airing them; washing the floors, walls, ceilings, wood-work, and all wooden articles of furniture with a strong solution of carbolic acid, and by burning all cloth goods (bedding, curtains, carpets, etc.) that were in the least infected.

